# Acetabular Revision Surgery with Tantalum Trabecular Metal Acetabular Cup for Failed Acetabular Cage Reconstruction with Bone Allografts: A Retrospective Study with Mid- to Long-Term Follow-Up

**DOI:** 10.3390/jcm11123428

**Published:** 2022-06-15

**Authors:** Chen-Heng Hsu, Chih-Chien Hu, Chih-Hsiang Chang, Yu-Han Chang, Hsin-Nung Shih, Chun-Chieh Chen

**Affiliations:** 1 Department of Orthopedic Surgery, Chang Gung Memorial Hospital, Taoyuan 333423, Taiwan; mp1796@cgmh.org.tw (C.-H.H.); r52906154@cgmh.org.tw (C.-C.H.); 8802032@cgmh.org.tw (C.-H.C.); yhchang@cgmh.org.tw (Y.-H.C.); aronc@cgmh.org.tw (H.-N.S.); 2 Bone and Joint Research Center, Chang Gung Memorial Hospital, Taoyuan 33305, Taiwan; 3 College of Medicine, Chang Gung University, Taoyuan 33302, Taiwan

**Keywords:** tantalum trabecular metal acetabular cup, failed cage reconstruction, revision hip arthroplasty

## Abstract

**Background**: Acetabular cage reconstruction with bone allografts is among the successful strategies to deal with massive acetabular bone loss. However, the nonbiological fixation nature of cages can compromise long-term success. Tantalum trabecular metal acetabular cups (TM cups) have been used in acetabular revision surgery because of their increased initial stability and good bone ingrowth features. This study was performed to determine whether the bone stock of the acetabulum is enough to support a hemispheric TM cup after failed cage reconstruction with bone allografts. **Methods**: We retrospectively reviewed patients who received acetabular revision surgery with TM cups after failed cage reconstruction with bone allografts from 2006 to 2017. There were 12 patients (5 males and 7 females) included in this study, with a mean age of 61.5 years (38 to 81) at the time of re-revision surgery. The mean follow-up after re-revision surgery was 8.6 years (2.6 to 13.3). The endpoint was defined as the aseptic loosening of the TM cup and reoperation for any causes. The change in bone stock of the acetabulum between index revision and re-revision was assessed according to the Gross classification for acetabular bone loss. **Results**: One patient died after eight years of follow-up of a cause not related to hip surgery. Two patients received two-stage revision arthroplasty due to PJI after 3.2 and 9.4 years of follow-up, respectively. The bone stock of the acetabulum was significantly improved between index revision and re-revision surgery (*p* < 0.0001). The Kaplan–Meier survivorship was 100% with aseptic loosening as the endpoint and 90% and 75% at five- and ten-year follow-up, respectively, with reoperation for any reason as the endpoint. Even cage reconstruction with bone allografts will fail eventually, and the bone stock of the acetabulum will improve after union and incorporation between host bone and allografts. The restored bone stocks will facilitate further revision surgery with hemispheric TM cups. The biological fixation between host bone and tantalum trabecular metal can provide longstanding stability of the TM cup. **Conclusions**: The results of our study offer a viable option for patients with failed cage reconstruction with bone allografts.

## 1. Background

Total hip arthroplasty (THA) is one of the most successful orthopedic interventions. However, because of its popularity, an increasing number of patients undergoing total hip arthroplasty require revision surgery. The main reasons for implant failure in 71% of cases are aseptic loosening and associated osteolysis [1]. In addition, acetabular bone loss after THA can be a serious problem in revision surgery, as bone deficiencies may limit reconstructive options, increase the difficulty of operation, and necessitate autogenous or allogenic bone grafting.

Treatment options for different types of acetabular bone defects have been well-established. Most patients can undergo reconstruction with uncemented cups with screws with or without morselized bone grafts. However, severe acetabular bone defects might require structural grafts, a cup with or without augments, a cage, or a cup cage, depending on the type of bone defect.

Cage reconstruction is used in massive bone defects in which an uncemented porous cup cannot achieve stable fixation or biologic fixation is unlikely, as well as in cases of pelvic discontinuity [2]. However, cages have high failure rates in mid- to long-term follow-up due to a lack of biologic fixation [3,4]. Although the cages themselves will fail eventually, allografts are protected by the cages; thus, grafts can restore the bone stock of the acetabulum [4,5,6]. Furthermore, the downgrading of the acetabular bone defect may allow for the use of an uncemented hemispheric cup in revision surgery when the cage fails. Therefore, we aimed to determine whether the acetabulum bone stock is sufficient to support hemispheric cups and achieve longstanding biological fixation after the cage is loosened.

Trabecular metal (TM, Zimmer Biomet, Warsaw, Indiana) is made of highly porous tantalum. It facilitates bone ingrowth and initial stability, owing to its low modulus of elasticity and high frictional characteristics [7,8,9]. TM cups have been demonstrated to lead to successful clinical outcomes and survivorship in revision hip surgery [10]. In a previous study, we used structural allografts with a TM cup to treat Paprosky type III defects in revision THA with good clinical outcomes [11].

Because of the bioactive nature and bone ingrowth properties of TM [10], we suspect that TM cups are suitable for patients who require acetabular revision surgery due to loosened cages. The purpose of this study was to report the mid- to long-term results of using trabecular-metal-coated hemispherical cups (TM cups) to salvage failed cages in acetabular revision surgery.

## 2. Methods

We retrospectively reviewed the rTHA register database of our institution and identified 2831 patients who had received rTHA in our institution from 2006 to 2017. We included patients who received acetabular revision surgery with TM cups due to aseptic loosening of cages. The first revision surgery using cage reconstruction with bone allografts was termed “index revision”, and the following revision surgery using a TM cup due to aseptic loosening of the cage was termed “re-revision.”

### 2.1. Patients

Twelve such patients (twelve hips), including five men and seven women, were included in this study. The mean age of the patients at index revision was 56.1 ± 14.7 years and 61.5 ± 14.6 years at re-revision. Patient demographics are summarized in Table 1.

Acetabular bone defects at the time of index revision and re-revision were classified based on pre-operative radiographs and intraoperative findings according to the Paprosky classification [12] and the Gross classification for acetabular bone loss [13]. Briefly, Paprosky type I defects are characterized by minimal bone loss, and intact dome, presented posterior wall and teardrop, and an uninvolved medial wall. Type II defects are characterized by a distorted acetabular hemisphere, a destructed dome and/or medial wall, and preserved anterior and posterior columns. Type III defects are characterized by severe bone loss, a destroyed acetabular rim, and supporting structures [12]. The Gross classification was defined as follows: type I: no significant loss of bone stock; type II: contained loss of bone stock (cavitary); type III: uncontained loss of bone stock involving less than 50% of the acetabulum (posterior column ± anterior column); type IV: uncontained loss of bone stock involving more than 50% of the acetabulum (segmental loss affecting both columns); and type V: pelvic discontinuity with uncontained loss of bone stock [13].

### 2.2. Surgical Procedure

**Figure 1:**
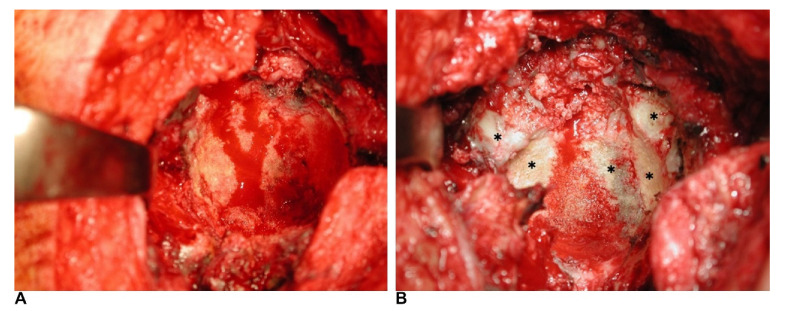
(**A**) The host bone of acetabulum was bleeding after reaming in re-revision surgery, indicating revascularization of allografts. (**B**) The allografts, which were implanted in index revision, had non-vascularized areas (marked with *) during re-revision surgery.

An anterolateral approach in lateral decubitus position was chosen for all patients in index revision and re-revision surgery. Bone allografts were obtained from the bone bank of our institution. The bone bank protocol, which consisted of donor selection, harvesting, and processing of the allograft, followed the recommendations of the Musculoskeletal Council of the American Association of Tissue Banks. During index revision surgery, the bone defects of the acetabulum were filled with structural and morselized fresh-frozen bone allografts after removal of the previous implant, debridement, and reaming to bleeding host bone. When the reasonable hip center was restored by bone grafting, the cage was placed within the acetabulum and affixed with screws. After the cage was secured, a PE liner was fixed to the cage in the appropriate orientation using bone cement. In re-revision surgery, after removing the failed cage and debridement, the acetabulum was reamed to bleeding host bone (Figure 1A). The acetabular bone defects were assessed, and structural and/or morselized bone grafts were applied to the segmental or cavity bone defects. Then, a TM cup was press-fitted to the acetabulum.

### 2.3. Outcome Assessment

The Gross classification was used to quantify the restoration of bone stock between index revision and re-revision surgery. The modified Harris hip score [14] was used to assess clinical status. All patients were followed routinely (6 weeks, 3 months, 6 months, and 1 year post-op and annually thereafter). In addition, we retrospectively reviewed medical records and radiographs, including an anteroposterior (AP) pelvis view and lateral hip view. Osseointegration of the TM cups after re-revision surgery was assessed according to the classification proposed by Moore et al. [15], which includes five radiographic signs: (1) absence of radiolucent lines, (2) presence of a superolateral buttress, (3) medial stress shielding, (4) radial trabeculae, and (5) an inferomedial buttress. Loosening of TM cups was defined as >3 mm horizontal or vertical migration, a change in the angle of inclination >5°, and fewer than two signs of osteointegration [15].

### 2.4. Statistical Analysis

All statistical analyses were performed with MedCalc Statistical Software version 19.2.5 (MedCalc Software Ltd., Ostend, Belgium; https://www.medcalc.org; accessed on 27 July 2020). Kaplan–Meier survival analysis was used to estimate the survivorship of the TM cup in re-revision surgery by two endpoints: (1) aseptic loosening and (2) reoperation for any reason. Restoration of acetabular bone loss was assessed using the Student’s paired *t*-test. A *p*-value of <0.05 was considered statistically significant.

## 3. Results

**Figure 2:**
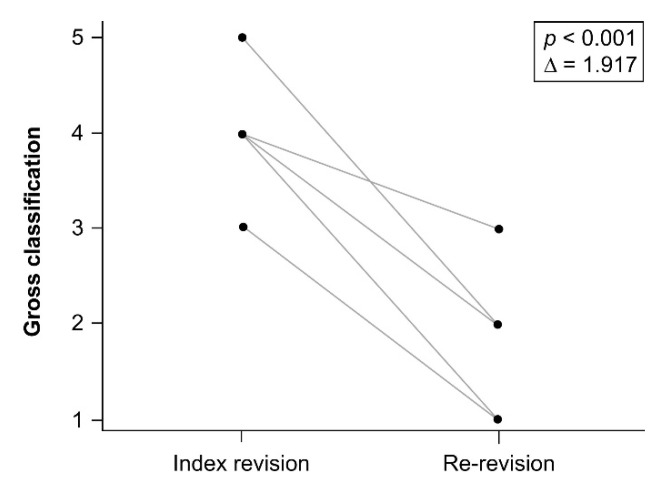
Acetabulum bone loss significantly decreased between index revision and re-revision according to Gross classification (*p* < 0.001).

**Figure 3:**
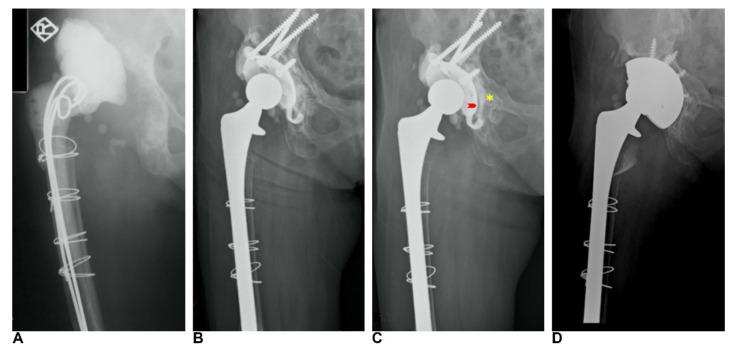
(**A**) Paprosky type IIIA acetabular bone defect after operation for periprosthetic joint infection. (**B**) Reconstruction of acetabulum bone loss with cage and bone allografts. (**C**) Cage failure eight years after index revision. Radiolucent line between cage and host bone (arrow), with union of the host bone and allografts (*) were shown. (**D**) The TM cup remained stable after nine years of follow-up after re-revision surgery.

**Figure 4:**
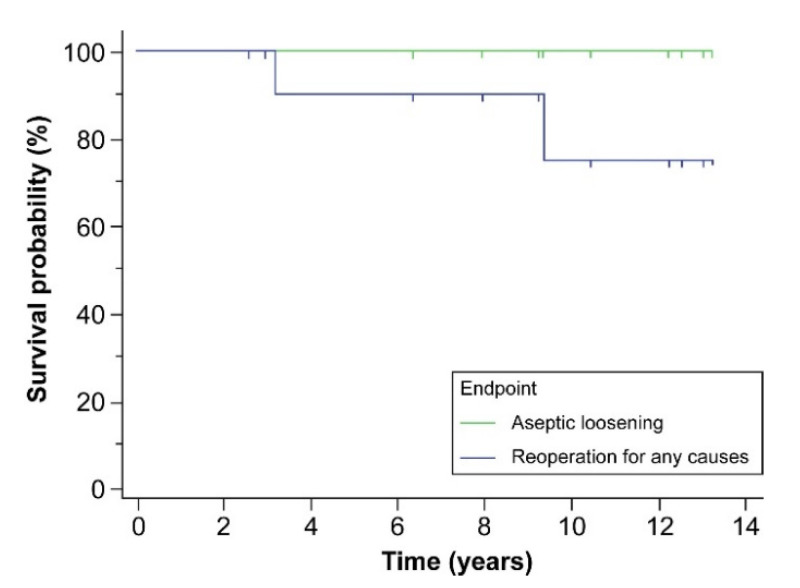
The Kaplan–Meier survivorship was 100% with aseptic loosening as the endpoint, and 90% and 75% at five- and ten years, respectively, with reoperation for any reason as the endpoint.

One patient died after eight years of follow-up for reasons unrelated to hip surgery, but the TM cup was stable at her last follow-up. Two patients suffered from hematogenous periprosthetic joint infection (PJI) 3.2 years and 9.4 years following re-revision surgery, respectively. These two patients received two-stage revision arthroplasty for PJI at the time of infection. The TM cups were stable during first stage surgery for PJI. The mean interval of index revision and re-revision of the twelve studied patients was 5.4 ± 3.4 years. At the time of index revision, there were five cases of Paprosky type IIIA defects, six type IIIB defects, and one case of pelvic discontinuity. At re-revision, there was one case of Paprosky type I, four type IIA, four type IIB, two type IIC, and one type IIIA defect. Overall, bone defects were downgraded in re-revision surgery. Bone defects at index revision and re-revision surgery according to the Gross classification are shown in Table 2. The bone stock of the acetabulum was significantly restored at the time of re-revision surgery (*p* < 0.001) according to the Gross classification (Figure 2). The mean follow-up time after re-revision surgery was 8.6 ± 4 years (Figure 3). With aseptic loosening defined as the endpoint, the Kaplan–Meier survivorship was 100%. When reoperation for any reason was set as the endpoint, the Kaplan–Meier survivorship at five- and ten year was 90% and 75%, respectively (Figure 4). There were no complications, such as dislocations, sciatic nerve palsy, or surgical site infection, after index revision or re-revision surgery. The mean modified Harris hip score was 85.6 ± 4.1 at the latest follow-up after re-revision surgery.

## 4. Discussion

Acetabular revision surgery with extensive acetabular bone defects is challenging and technically demanding. Massive bony deficiencies can arise from osteolysis after polyethylene wear, destruction of the bone by loosening of an acetabular component, repeated revision of the acetabulum, or extended debridement for PJI. When dealing with massive acetabular bone loss, such as Paprosky type III acetabular defect, structural allografts not only restore bone stock but also support new acetabular components [16]. Garbuz et al. reported a high failure rate of acetabular revisions if structural allografts supported >50% of the cup at a mean follow-up of 7 years, and more than half of failed cases showed both prosthesis and allograft failure [5]. Several previous studies have reported that reconstruction of massive acetabular bone defects using a cage with allografts provided good initial stability [17,18]. However, the inability to achieve biologic fixation at the cage–bone interface can compromise the chances of long-term success [3,4]. The cage will eventually fail because of its nonbiological fixation.

Allogenic bone grafts are biocompatible scaffolds. Revascularization, resorption, and appositional bone growth will occur slowly [16]. During acetabular revision surgery, the cage can protect the bone grafts, and the bone stock will restore over time through the union and incorporation between host bone and bone grafts [3]. In our study, bone base bleeding was noted after removing the loosened cages and reaming the acetabulum in re-revision surgery. It indicated the host bone had incorporated with the allografts, which were implanted during index revision surgery. This bled living bone, remodeling from allografts, thus renovating the bone stock of the acetabulum.

The new bone stock can downgrade the bone defects of the acetabulum and facilitate re-revisions. Abolghasemian et al. reported that 48% of patients with Gross type III or higher acetabular bone defects at index revision were downgraded to Gross type I or II defects at re-revision [19]; moreover, 60% of hips could use hemispherical cups in re-revision surgery. In our study, all acetabular bone defects were restored through re-revision surgery, according to the Gross classification. The reconstitution of acetabulum bone stock provided an opportunity to use cementless hemispherical components in re-revision surgery.

As shown in Figure 1B, although the allografts had united with the host bone, the bone was not all revascularized (no bleeding area, marked as *), and allograft had been absorbed. A revascularized allograft predisposes to weakening and loosening of the structure [20]. Although there were Gross type I or II acetabular bone defects in re-revision surgery, the bleeding host bone may not be strong enough to support a conventional uncemented cup [10].

The more viable bony base and the better overall bone environment of the acetabulum improve uncemented cup fixation and stabilization. In addition, systemic pharmacological therapy to improve bone metabolism may help improve the revascularization and osseointegration of allogenic bone grafts implanted during index revision surgery. Teriparatide (parathyroid hormone [PTH1-34]) is an anabolic agent reported to increase bone formation by stimulating osteoblast differentiation, function, and survival [21,22]. The application of teriparatide in fractures of animal models resulted in increased callus volume, as well as improved mineralization and mechanical strength [23]. Application of teriparatide aid in the incorporation of allogenic bone grafts and contribute to acetabular implant stability; however, additional research is needed.

TM is made of tantalum, a highly porous biomaterial (porosity of 70–80%) in 3D architecture. One animal model study reported bone ingrowth within two weeks, and substantial ingrowth occurred within four weeks [7]. Other studies have reported that TM cups are suitable for acetabular revision surgery with massive bone loss, even with less than 50% of the acetabulum host bone [10,11,24]. For example, Kosashvili et al. reported 15 patients receiving non-buttressed TM cup reconstruction for failed acetabular cages with a mean follow-up time of 48.3 months (range, 24–72 months). Of these 15 patients, 12 did not experience cup loosening at the end of the follow-up period [25]. In the current study, we observed no radiological failures due to aseptic loosening after re-revision surgery at a mean follow-up time of 8.7 years, and only two hips failed due to infection after 3.2- and 9.4-years follow-up, respectively. In addition, TM cups provide improved mechanical stability and a more favorable environment for bone incorporation [26,27]. For these reasons, we used TM cups in re-revision surgery after failed cage reconstruction with bone allografts in our clinical practice with good results.

There are some limitations to our study that need to be addressed. First, the number of patients in this study was relatively small, despite the high volume of referrals to our institution. Additionally, we were not able to compare this technique to other revision techniques, as this was a retrospective study and not a randomized study; thus, there may have been a selection bias. Finally, we used TM cups to salvage failed cages based on the evaluation of pre-operative radiographs and assessed acetabular bone defects and the quality of bleeding living bone intraoperation. However, our study suggests that TM cup reconstruction is a viable option for re-revision surgery after failed cage reconstruction with bone allografts.

## 5. Conclusions

The results of the present study suggest that using cages in combination with allografts in acetabular revision surgery with massive bone loss can restore acetabular bone stock. Although the cages will eventually fail, the reconstituted bone stock can help with future revisions. TM cups are a reliable option and a relatively more accessible procedure for re-revision surgery after failed cage reconstruction with bone allografts. The benefit of TM cups for biological fixation include long-lasting construction after re-revision surgery.

## Figures and Tables

**Table 1:** Patient demographics.

Variable	All Hips (N = 12)
Sex, n	
Male	5
Female	7
Mean age at index revision surgery, years (SD)	56.1 (14.7)
Mean age at re-revision surgery, years (SD)	61.5 (14.6)
Mean interval between index revision and re-revision, years (SD)	5.4 (3.4)
Mean follow-up after re-revision, years (SD)	8.6 (4)
**Reason for index revision surgery, n**	
Loose acetabular component	9
Second-stage reimplantation for infection	3
**Paprosky classification for index revision, n**	
IIIA	5
IIIB	6
Pelvic discontinuity	1
**Paprosky classification for re-revision, n**	
I	1
IIA	4
IIB	4
IIC	2
IIIA	1

**Table 2:** Gross classification of acetabular bone loss at index and re-revision surgery.

	Defect (n)				
Surgery	Type I	Type II	Type III	Type IV	Type V
Index revision			1	10	1
Re-revision	4	3	5		

## Data Availability

The data presented in this study are available in supplementary material.

## Supplementary Materials

The following supporting information can be downloaded at: https://www.mdpi.com/article/10.3390/jcm11123428/s1.
